# Controllable oxidative stress and tissue specificity in major tissues during the torpor–arousal cycle in hibernating Daurian ground squirrels

**DOI:** 10.1098/rsob.180068

**Published:** 2018-10-10

**Authors:** Yanhong Wei, Jie Zhang, Shenhui Xu, Xin Peng, Xia Yan, Xiaoyu Li, Huiping Wang, Hui Chang, Yunfang Gao

**Affiliations:** 1 Key Laboratory of Resource Biology and Biotechnology in Western China, College of Life Sciences, Northwest University, Ministry of Education, Xi'an 710069, People's Republic of China; 2 School of Basic Medical Sciences, Ningxia Medical University, Yinchuan 750004, People's Republic of China

**Keywords:** hibernation, oxidative stress, ROS, antioxidant enzymes, antioxidant defence

## Abstract

Mammalian hibernators experience repeated hypoxic ischaemia and reperfusion during the torpor–arousal cycle. We investigated levels of oxidative stress, antioxidant capacity, and the underlying mechanism in heart, liver, brain and kidney tissue as well as plasma during different periods of hibernation in Daurian ground squirrels (*Spermophilus dauricus*). Our data showed that the levels of hydrogen peroxide significantly increased in the heart and brain during late torpor (LT) compared with levels during the summer active (SA) state. The content of malondialdehyde (MDA) was significantly lower during interbout arousal (IBA) and early torpor (ET) than that during SA or pre-hibernation (PRE), and MDA levels in the LT brain were significantly higher than the levels in other states. Superoxide dismutase 2 protein levels increased markedly in the heart throughout the entire torpor–arousal cycle. Catalase expression remained at an elevated level in the liver during the hibernation cycle. Superoxide dismutase 1 and glutathione peroxidase 1 (GPx1) expression increased considerably in all tissues during the IBA and ET states. In addition, the activities of the various antioxidant enzymes were higher in all tissues during IBA and ET than during LT; however, GPx activity in plasma decreased significantly during the hibernation season. The expression of p-Nrf2 decreased in all tissue types during IBA, but significantly increased during LT, especially in liver tissue. Interestingly, most changed indicators recovered to SA or PRE levels in post-hibernation (POST). These results suggest that increased reactive oxygen species during LT may activate the Nrf2/Keap1 antioxidant pathway and may contribute to the decreased MDA levels found during the IBA and ET states, thereby protecting organisms from oxidative damage over the torpor-arousal cycle of hibernation. This is the first report on the remarkable controllability of oxidative stress and tissue specificity in major oxidative tissues of a hibernator.

## 1. Introduction

Hibernation is a distinctive strategy implemented by certain mammals to survive under conditions of low environmental temperature and food scarcity during winter and can be characterized by repeated torpor–arousal cycles. This succession of entry into torpor, deep torpor, arousal and aroused state is called a hibernation bout. The entire hibernation season is a sequence of 10–20 such bouts, depending on the duration of the season and length of the deep torpor periods [1]. Recurring periods of torpor commonly last one to two weeks in small mammalian hibernators, interspersed with brief spontaneous arousal resulting in euthermic rewarming (less than 24 h) [2]. During torpor, many physiological functions are significantly suppressed compared with those under normal euthermic conditions; for example, substantial decreases in metabolic rate (1%–5%), declines in body temperature (Tb) to near ambient temperature (as low as 1°C–5°C), and considerable drops in blood flow, heart rate and oxygen consumption [3–5]. However, initiation of interbout arousal from torpor is accompanied by the rapid recovery of many of these physiological functions, including the return of normal Tb (35°C–38°C), oxygen consumption and metabolic rate [2,5]; for example, during arousal from torpor in thirteen-lined ground squirrels (*Spermophilus tridecemlineatus*), oxygen consumption increased by 35-fold in about 2 h as Tb rose from 3°C to 31°C [4].

The torpor–arousal cycle of mammalian hibernators can be considered as an ischaemia–reperfusion cycle [6,7]. During interbout arousal, rapid reperfusion of blood flow to all tissues is accompanied by increased mitochondrial respiration and oxygen levels, which are known to cause elevated generation of reactive oxygen species (ROS) in mammals. As by-products of biochemical metabolic reactions, ROS are generated due to incomplete reduction of molecular oxygen in the electron transport chain of mitochondria, and include superoxide radicals 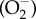, hydroxyl radicals (OH) and hydrogen peroxide (H_2_O_2_) [8,9]. Adequate amounts of ROS are considered as ‘second messengers’ in intracellular signalling and regulation, whereas excessive amounts of ROS can oxidize biomolecules such as lipids, carbohydrates, proteins and DNA, thereby impairing normal cellular functions and promoting cell death [10,11]. Theoretically, sharply increased Tb as well as oxygen consumption and mitochondrial respiration will result in the elevated generation of ROS in tissue [12–14]. Malondialdehyde (MDA) and conjugated diene, indices of oxidative damage of lipids, can cause toxic stress in cells. Previous studies have shown that MDA in plasma and erythrocyte membranes of black bears (*Ursus americanus*) and conjugated diene levels in the intestines of thirteen-lined ground squirrels significantly increase during hibernation, implying increased oxidative stress during this state [15,16]. However, Ma *et al*. [6] reported no evidence of oxidative injury in the brains of hibernating Arctic ground squirrels (*S. parryii*) after hibernation, including no cellular stress, inflammatory response or neuronal pathology [6]. Previous studies in our laboratory on hibernating Daurian ground squirrels (*Spermophilus dauricus*) found no apoptosis in skeletal muscle by TUNEL assay [17]. Thus, although oxidative stress does occur in some tissues, no oxidative damage has been found during prolonged periods of hibernation. Small hibernators are, therefore, an interesting anti-oxidative damage model.

Enhanced antioxidant defence capacity is very important for scavenging excess ROS and maintaining the redox homeostasis of cells in hibernating mammals. Further studies suggest that hibernators rely on the upregulation of multiple antioxidant enzymes and low molecular weight antioxidant proteins to avoid oxidative stress during hibernation, including superoxide dismutase (SOD)1, SOD2, catalase (CAT), glutathione peroxidase (GPx), peroxiredoxin (Prdx), thioredoxin 1 (Trx1) and ascorbate [7,18–24]. For example, the upregulation of Prdx, Trx1 and SODs in brown adipose tissue, white adipose tissue and heart tissue has been shown to contribute to antioxidant defence during hibernation in thirteen-lined ground squirrels [22,23]. Furthermore, SOD1, SOD2, CAT, glutathione reductase (GSR), GPx1 and Trx2 are reported to increase significantly during interbout arousal in the brains of hibernating bats (*Myotis ricketti* and *Rhinolophus ferrumequinum*) [24]. Undoubtedly, the upregulation of multiple antioxidant proteins can effectively scavenge the overproduction of ROS, implying that the elevated antioxidant defence system may play a key role in coping with redox imbalance during hibernation.

The Nrf2 (nuclear factor erythroid 2 (NF-E2)-related factor 2)/Keap1 (Kelch-like ECH-associated protein 1) pathway is a prominent regulator of cytoprotective responses to oxidative stress [25,26]. Under basal conditions, Nrf2 is sequestered in the cytosol by a Keap1 homodimer, which facilitates the ubiquitination and proteasomal degradation of Nrf2. However, under conditions of oxidative stress, Nrf2 is released from Keap1, leading to phosphorylation and nuclear translocation. In the nucleus, phospho-Nrf2 heterodimerizes with small musculoaponeurotic fibrosarcoma (Maf) proteins and binds to the antioxidant response element (ARE), thereby activating the expression of a battery of antioxidant genes that respond to oxidative stress [27–29]. Well-known Nrf2-responsive antioxidant genes include SOD, CAT, GPx and NADH quinone oxidoreductase 1 (NQO1) [30,31]. These earlier studies have focused on changes in the antioxidant capacity of different animal species during hibernation using different tissues. However, systematic research is still lacking and, more importantly, the mechanism of antioxidant damage remains unknown. Changes in the levels of oxidative stress in repeated torpor–arousal cycles during hibernation are also unknown. Therefore, systematic assessments of the level of oxidative stress, the regulation of antioxidant defence capacity, and the underlying mechanism in major oxidative tissues in mammalian hibernators are essential, particularly the exploration of the mechanism for preventing ischaemia–reperfusion-induced oxidative injury during repeated torpor–arousal cycles, which is a fascinating and challenging topic.

We hypothesized that hibernating squirrels might adjust their antioxidant defence to prevent or diminish potential oxidative damage in major oxidative tissues during the torpor–arousal cycle; moreover, the differences in oxygen consumption and blood flow in tissues should result in differences in levels of oxidative stress. In the human body, blood flow in the heart is 60–90 ml (100 g min)^−1^ at resting state, while oxygen demand is 10-fold higher than the average oxygen demand of all body tissues and 4.5-fold higher than that of skeletal muscle [32]. Furthermore, blood flow of the brain accounts for 15%–20% of total blood volume, whereas oxygen consumption accounts for approximately 25% of total oxygen consumption of the entire body [32]. Therefore, heart and brain tissues are more susceptible and more easily damaged than other tissues due to oxidative stress induced by hypoxic ischaemia and reperfusion [32]. The liver is an important detox apparatus of the body, whereas the kidney is a major excretory organ involved in the regulation of blood pressure, erythrocyte production and calcium metabolism and is closely related to oxidative stress [33]. Therefore, to test the above hypothesis, we used heart, liver, brain, kidney and plasma from hibernating Daurian ground squirrels to examine the time course of H_2_O_2_ and MDA content in these tissues over the entire hibernation period. The expression levels and antioxidant activities of the SOD1, SOD2, CAT and GPx1 enzymes as well as total antioxidant capacity (TAC) during the various states of hibernation were further determined. We also explored the molecular mechanisms involved in the regulation of antioxidant responses during hibernation by measuring the expression of the Nrf2/Keap1 pathway.

## 2. Material and methods

### 2.1. Animal care and experimental groups

All animal experiments were approved by the Laboratory Animal Care Committee of the P. R. China Ministry of Health. Daurian ground squirrels were captured in mid-June from the Wei Nan region of the Shaanxi Province in China. Some squirrels were used immediately as the summer active (SA) control. The remaining squirrels were housed in the laboratory at 18°C–25°C and provided with food and water *ad libitum* until they entered and finished the pre-hibernation phase, as described previously [34,35]. Tb was measured using thermal imaging with a visual thermometer (Fluke VT04 Visual IR Thermometer, USA) throughout the hibernation season (twice a day, 08.00 and 20.00). For the experimental hibernation group, samples were retrieved under the following conditions/states (i) pre-hibernation group (PRE): squirrels had not entered torpor by late September and showed Tb values of 36°C–38°C; (ii) early torpor group (ET): after two months hibernation (70 ± 8 days), animals entered into a new hibernation bout with Tb maintained at 5°C–8°C for less than 24 h; (iii) late torpor group (LT): after two months hibernation (65 ± 5 days), animals entered into a new hibernation bout and were in continuous torpor for at least 5 days with a stable Tb of 5°C–8°C; (iv) interbout arousal group (IBA): after two months hibernation (68 ± 5 days), animals entered into a new hibernation bout and aroused spontaneously with a Tb returning to 34°C–37°C for less than 12 h; and (v) post-hibernation group in spring (POST): animals fully and spontaneously awakened from hibernation (118 ± 14 days) and maintained a Tb of 36°C–38°C for 3 days in March of the following year. In total, 192 adult and non-reproductive Daurian ground squirrels were weight-matched and randomly grouped into six groups (*n* = 8 per organ in each group). The female to male ratio was usually 3 : 1 per group.

Note, ET and LT were both hibernating groups, but their durations of torpor were different. We measured the levels of oxidative stress and antioxidant defence with the extension of hibernation time during a hibernation bout by comparing the levels for ET and LT. This was also useful for evaluating the role of periodic interbout arousal (IBA) in antioxidant defence during the hibernation season. Squirrels maintained stable torpor with a low Tb (5°C–8°C) for 7–10 days during a hibernation bout after experiencing two months of hibernation. Thus, to ensure the individual changes in each LT group were consistent, our sampling time was set to 5 days with a stable Tb of 5°C–8°C.

### 2.2. Tissue acquisition

All animals were anaesthetized with 90 mg kg^−1^ sodium pentobarbital. After anaesthetization, the animals were surgically opened at the chest and one tissue type was collected immediately, after which the wet mass was recorded, and the tissue sample was rapidly frozen in liquid nitrogen. To avoid post-traumatic stress due to ischaemia and pain during the operation, the tissue samples for the study of oxidative stress were not obtained from the same squirrel. At the end of surgical intervention, all animals were sacrificed by an overdose injection of sodium pentobarbital. All heart, liver, brain, kidney and plasma samples were subsequently stored at −80°C until further processing.

### 2.3. Quantification of reactive oxygen species

Direct detection of ROS and other free radicals is difficult because these molecules are short-lived and highly reactive in a nonspecific manner [10]. Ongoing levels of ROS are therefore generally analysed by measurement of secondary products, such as derivatives of amino acids (e.g. H_2_O_2_) and lipid peroxidation (e.g. MDA). As H_2_O_2_ is an ROS and ROS can degrade polyunsaturated lipids to form MDA, thus causing toxic stress in cells, both MDA and H_2_O_2_ levels in tissue (heart, liver, brain and kidney) and plasma samples from hibernating Daurian ground squirrels were measured.

Frozen heart, liver, brain and kidney samples (approx. 0.1 g) were homogenized in 0.9 ml of PBS (137 mM NaCl, 2.7 mM KCl, 2 mM KH_2_PO_4_ and 10 mM Na_2_HPO_4_) at 4°C with a Scientz-48 high-throughput tissue grinder (Scientz Biotechnology, China). After the homogenates were centrifuged at 3000 r.p.m. for 15 min at 4°C, the resultant supernatants were used for biochemical analyses. The protein concentrations in the supernatants were determined using a Pierce™ BCA protein quantitation kit (Thermo Fisher Scientific, Rockford, IL, USA).

The MDA concentration in the homogenates was determined using malondialdehyde (MDA) assay kits (Nanjing Jiancheng Bioengineering Institute, Nanjing, China) based on thiobarbituric acid (TBA) reactivity. As lipid peroxidation forms MDA and 4-hydroxynonenal (4-HNE), their levels are considered measures of oxidative damage. MDA readily reacts with TBA to generate MDA-TBA adduct (a type of TBA reactive substance, TBARS), which can be quantified colorimetrically. Briefly, after mixing trichloroacetic acid with each homogenate and centrifuging at 3000 r.p.m. for 15 min at 4°C, a supernatant was obtained, to which TBA was then added. The developed red colour of the resulting reaction was measured at 532 nm with a spectrophotometer (Shimadzu UV-2550, Japan). Other procedures were carried out following the manufacturer's protocols. The MDA content was calculated according to the formula: MDA content (nmol mg^−1^ protein) = (absorbance of sample − absorbance of standard blank sample)/(absorbance of standard sample − absorbance of standard blank solution) × sample protein concentration (mg protein ml^−1^).

The concentration of H_2_O_2_ in the tissue samples was measured using a hydrogen peroxide (H_2_O_2_) assay kit in accordance with the manufacturer's protocols (Nanjing Jiancheng Bioengineering Institute, Nanjing, China). The H_2_O_2_ was bound with molybdenic acid to form a complex, which was measured at 405 nm with a spectrophotometer (Shimadzu UV-2550, Japan). The concentration of H_2_O_2_ was then calculated according to the formula: H_2_O_2_ content (mmol l^−1^) = (absorbance of sample − absorbance of blank solution)/(absorbance of standard sample − absorbance of standard blank solution) × concentration of standard sample × dilution multiple of sample.

### 2.4. Antioxidant activity assay

The activities of SOD, CAT and GPx in the tissue homogenates were assessed using superoxide dismutase (SOD), catalase (CAT) and glutathione peroxidase (GPx) assay kits (Nanjing Jiancheng Bioengineering Institute, Nanjing, China), respectively, according to the manufacturer's protocols. SOD activity was based on the auto-oxidation of hydroxylamine, with the developed blue colour then measured at 550 nm. The decomposition of H_2_O_2_ by CAT was stopped by the addition of ammonium molybdate. The remaining H_2_O_2_ was then reacted with ammonium molybdate to generate a pale-yellow complex, which was measured at 405 nm. The activity of CAT was then calculated. A series of enzymatic reactions was activated by GPx in the homogenate, leading to the conversion of GSH (reduced glutathione) to oxidized glutathione (GSSG). The change in absorbance during this conversion was recorded spectrophotometrically at 412 nm (Shimadzu UV-2550, Japan). The TAC in tissue homogenates was detected using a total antioxidant capacity (TAC) assay kit (Nanjing Jiancheng Bioengineering Institute, Nanjing, China).

### 2.5. Western blotting

Western blotting was undertaken as described previously [35]. Total protein was extracted from the brain, heart, liver and kidney tissue samples and homogenized with 1 mM RIPA lysis buffer (Heart, Xi'an, China) and 10 µl ml^−1^ protease inhibitor cocktail (Heart, Xi'an, China). The supernatants were collected after centrifugation at 15 000 r.p.m. for 15 min at 4°C. Soluble protein concentration was assayed using a Pierce™ BCA protein quantitation kit (Thermo Fisher Scientific, Rockford, IL, USA). The samples were then adjusted to a final protein concentration of 5 µg µl^−1^ by the addition of a small volume of homogenizing buffer. The aliquots were combined 1 : 4 (v : v) with 1× SDS loading buffer and then boiled. The final protein samples at a concentration of 2.5 µg µl^−1^ were stored at −80°C until use.

SDS-polyacrylamide gel electrophoresis and western blotting on polyvinylidene fluoride (PVDF) membranes were carried out with equal amounts of protein from various samples [35] with 10% gels (5% stacking gel), 20 µg of protein per well, and electrophoresis at 200 V for 45 min. After electrophoresis, the semi-dry transfer of proteins onto 0.45-µm PVDF membranes was conducted using a transfer buffer solution containing 25 mM Tris (pH = 8.5) at 120 V for 30–40 min. Following transfer, membranes were blocked with 5% (w : v) milk in 1× TBST (20 mM Tris-HCl, pH 7.6, 140 mM NaCl, 0.05% (v : v) Tween-20, 90% (v : v) ddH_2_O) for 2 h at 37°C, and then probed with primary antibodies at 4°C overnight. Primary antibodies against SOD1, SOD2, CAT, GPx1, Nrf2, Nrf2 (phospho S40) and Keap1 are listed in the electronic supplementary material, table S1. Membranes were then incubated with HRP-linked anti-rabbit IgG secondary antibody (1 : 5000 (v : v) dilution, Thermo Fisher Scientific, USA, A27014) in TBST for 2 h at room temperature. After washing with TBST (4 × 5 min), the membranes were visualized using enhanced chemiluminescence reagents (Thermo Fisher Scientific, USA, NCI5079) according to the manufacturer's protocols. Quantification analysis of the intensity of each band was performed using NIH Image J software.

### 2.6. Statistical analysis

One-way analysis of variance (ANOVA) and multiple comparison analyses were used to determine group differences, with LSD multiple comparison used when normality (and homogeneity of variance) was detected and ANOVA-Dunnett's T3 method used when no homogeneity was detected. SPSS 19.0 was used for all statistical tests. A *p*-value of less than 0.05 was considered statistically significant.

## 3. Results

### 3.1. Organ wet mass and organ-to-body mass ratio in tissue samples

**Figure 1.**
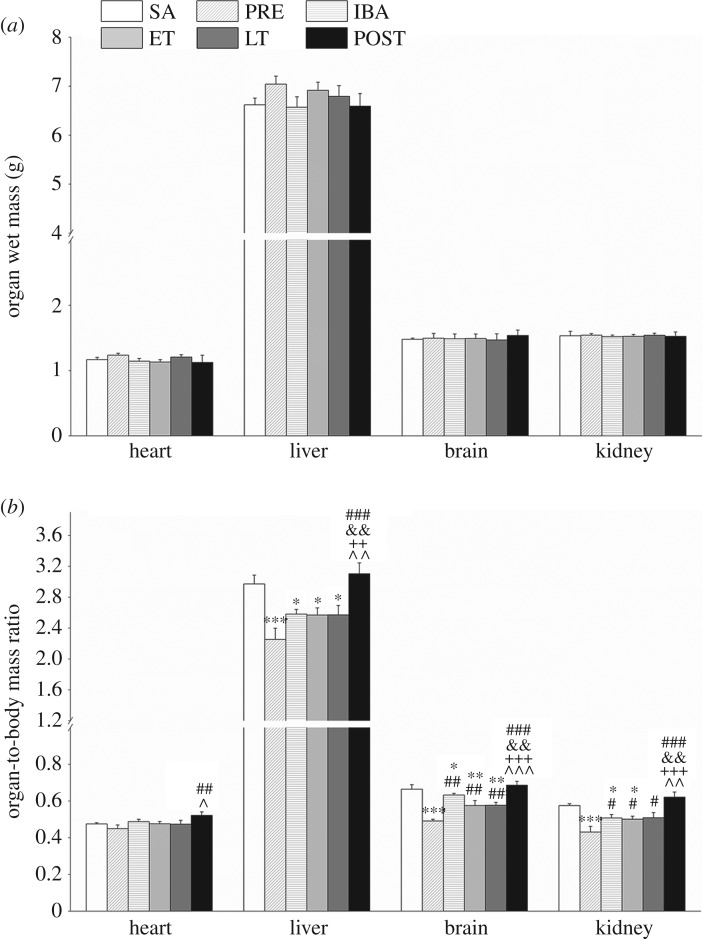
Effects of hibernation on body weight, organ wet weight and organ-to-body-mass ratio in organs of *S. dauricus* over the course of a torpor–arousal cycle. (*a*) Changes in organ wet weight of heart, liver, brain and kidney in different periods (*n* = 8, one-way ANOVA). (*b*) Changes in organ-to-body-mass ratio in heart, liver, brain and kidney in different periods (*n* = 8, one-way ANOVA). SA, summer active; PRE, pre-hibernation; IBA, interbout arousal; ET, early torpor; LT, late torpor; POST, post-hibernation. Data are means ± s.e; *p < 0.05, compared with SA; **p < 0.01 compared with SA; ***p < 0.001 compared with SA; ^#^*p* < 0.05, compared with PRE; ^##^*p* < 0.01, compared with PRE; ^###^*p* < 0.001, compared with PRE; ^&&^*p* < 0.01, compared with IBA; ^++^*p* < 0.01, compared with ET; ^+++^*p* < 0.001, compared with ET; ^^^*p* < 0.05, compared with LT; ^^^^*p* < 0.01, compared with LT; ^^^^^*p* < 0.001, compared with LT.

We first investigated the organ wet mass and organ-to-body mass ratio in different organs (heart, liver, brain and kidney) (figure 1*a*,*b*). No significant changes in heart, liver, brain or kidney wet mass were observed (figure 1*a*). The ratios of organ wet weight/body weight showed a significant decrease in the PRE, IBA, ET and LT groups compared with that in the SA group (figure 1*b*); however, the ratios recovered to SA levels in all organs after hibernation.

### 3.2. Reduced malondialdehyde and H_2_O_2_ levels during interbout arousal and early torpor in tissues of hibernating Daurian ground squirrels

**Figure 2.**
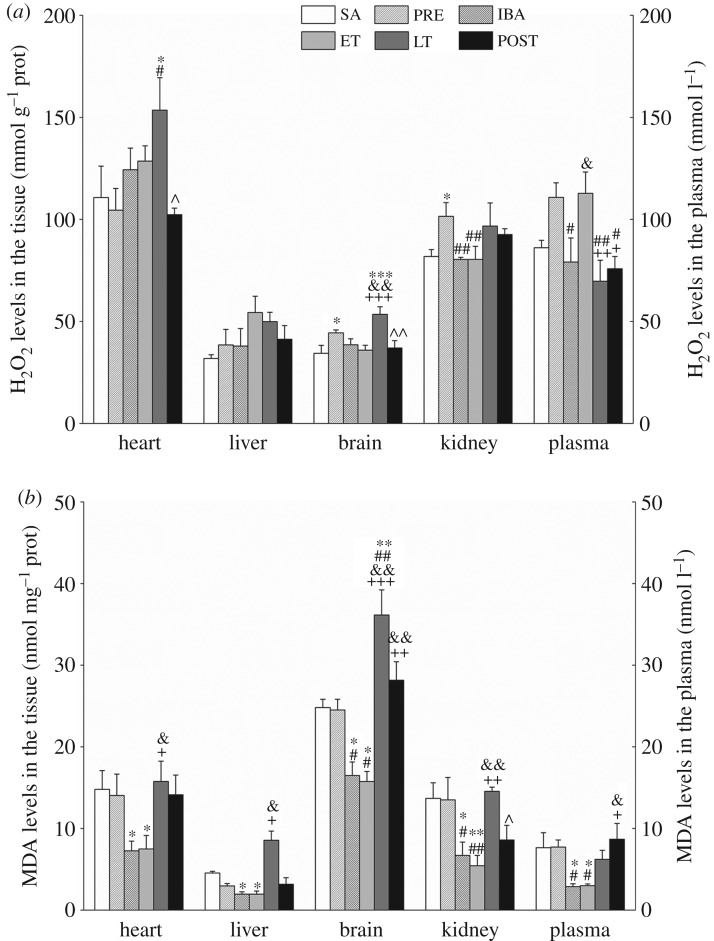
Levels of hydrogen peroxide (H_2_O_2_) and malondialdehyde (MDA) in tissue and plasma samples. (*a*) Changes in H_2_O_2_ levels in heart, liver, brain, kidney and plasma during different periods (*n* = 8, one-way ANOVA). (*b*) Changes in MDA levels in heart, liver, brain, kidney and plasma during different periods (*n* = 8, one-way ANOVA). SA, summer active; PRE, pre-hibernation; IBA, interbout arousal; ET, early torpor; LT, late torpor; POST, post-hibernation. Data are means ± s.e; *p < 0.05, compared with SA; **p < 0.01, compared with SA; ^#^*p* < 0.05, compared with PRE; ^##^*p* < 0.01, compared with PRE; ^&^*p* < 0.05, compared with IBA; ^&&^*p* < 0.01, compared with IBA; ^+^*p* < 0.05, compared with ET; ^++^*p* < 0.01, compared with ET; ^+++^*p* < 0.001, compared with ET; ^^^*p* < 0.05, compared with LT; ^^^^*p* < 0.01, compared with LT.

The content of H_2_O_2_ in the heart tissue increased significantly in the LT group compared with the SA and PRE groups (38.7%, *p* < 0.05; 46.9%, *p* < 0.05, respectively). However, no significant differences were observed among the different groups in regard to liver tissue. In the brain tissue, the content of H_2_O_2_ increased significantly by 29.2% (*p* < 0.05) in the PRE group and 55.5% (*p* < 0.001) in the LT group compared with the SA group; furthermore, during the torpor–arousal cycle, the H_2_O_2_ content in the LT group was higher than that in the IBA group (38.6%, *p* < 0.01) and ET group (48.8%, *p* < 0.001). In the kidney, H_2_O_2_ content in the PRE group increased by 24.1% (*p* < 0.05) relative to that in the SA group. In plasma, the content of H_2_O_2_ in the PRE and ET groups was slightly higher than that in the SA group. In addition, H_2_O_2_ content recovered to SA levels in all tissue and plasma samples after hibernation (POST group) (figure 2*a*).

The content of MDA in the heart decreased significantly in the IBA and ET groups compared with the SA group (51.0%, *p* < 0.05; 49.3%, *p* < 0.05, respectively). In the liver tissue, the level of MDA decreased markedly in the IBA and ET groups compared with the SA group (57.1%, *p* < 0.05; 53.3%, *p* < 0.05, respectively). In the brain tissue, the level of MDA decreased considerably in the IBA and ET groups compared with the SA group (33.6%, *p* < 0.05; 48.7%, *p* < 0.05, respectively), but increased significantly in the LT group relative to the SA group (45.7%, *p* < 0.01). In the kidney, MDA content decreased in the IBA and ET groups compared with the SA group (51.1%, *p* < 0.05; 60.4%, *p* < 0.01, respectively). In plasma, the content of MDA decreased significantly in the IBA and ET groups compared with the SA group (62.4%, *p* < 0.05; 60.9%, *p* < 0.05, respectively). Similar to the changes in H_2_O_2_ levels, the content of MDA recovered to normal levels in all tissue and plasma samples in the summer after hibernation (figure 2*b*).

### 3.3. Increased expression of antioxidant proteins during interbout arousal and early torpor but decreased expression during late torpor in Daurian ground squirrels

**Figure 3.**
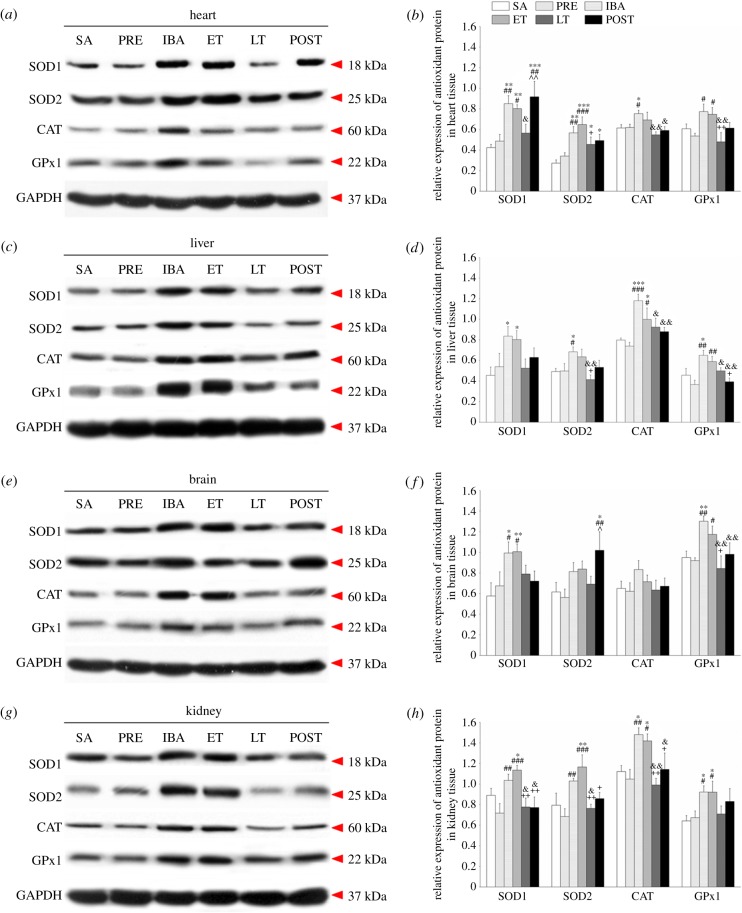
Changes in levels of SOD1, SOD2, CAT and GPx1 proteins in different tissues of *S. dauricus* over a torpor–arousal cycle. (*a*) Representative immunoblots of SOD1, SOD2, CAT and GPx1 in heart tissue during six hibernation periods. (*b*) Relative SOD1, SOD2, CAT and GPx1 protein expression in heart tissue. (*c*) Representative immunoblots of SOD1, SOD2, CAT and GPx1 in liver tissue during six hibernation periods. (*d*) Relative SOD1, SOD2, CAT and GPx1 protein expression in liver tissue. (*e*) Representative immunoblots of SOD1, SOD2, CAT and GPx1 in brain tissue during six hibernation periods. (*f*) Relative SOD1, SOD2, CAT and GPx1 protein expression in brain tissue. (*g*) Representative immunoblots of SOD1, SOD2, CAT and GPx1 in kidney tissue during six hibernation periods. (*h*) Relative SOD1, SOD2, CAT and GPx1 protein expression in kidney tissue. SA, summer active; PRE, pre-hibernation; IBA, interbout arousal; ET, early torpor; LT, late torpor; POST, post-hibernation. Values are means ± s.e., *n* = 8; *p < 0.05, compared with SA; **p < 0.01, compared with SA; ***p < 0.001, compared with SA; ^#^*p* < 0.05, compared with PRE; ^##^*p* < 0.01, compared with PRE; ^###^*p* < 0.001, compared with PRE; ^&^*p* < 0.05, compared with IBA; ^&&^*p* < 0.01, compared with IBA; ^+^*p* < 0.05, compared with ET; ^++^*p* < 0.01, compared with ET; ^^^*p* < 0.05, compared with LT; ^^^^*p* < 0.01, compared with LT.

To investigate antioxidant defences, we used western blotting to determine the expressions of four antioxidant proteins (SOD1, SOD2, CAT and GPx1) in heart, liver, brain and kidney tissue. The expressions of SOD1 and GPx1 increased markedly in all four tissues in the IBA and ET groups compared with the SA and PRE groups; SOD2 expression increased substantially in the heart throughout the entire torpor–arousal cycle, but increased significantly in the brain only in the POST group; furthermore, CAT expression remained at an elevated level in the liver during the hibernation cycle and increased significantly in the heart and kidney in the IBA group, but showed no obvious change in brain tissue (figure 3).

Compared with the SA group, SOD1 protein levels in the heart tissue of the IBA, ET and POST groups increased significantly by 100.8% (*p* < 0.01), 89.3% (*p* < 0.01) and 116.5% (*p* < 0.001), respectively. SOD2 expression maintained a higher level through the course of hibernation relative to the SA group, and increased by 107.3% (*p* < 0.01), 136.7% (*p* < 0.001), 66.3% (*p* < 0.05) and 79.9% (*p* < 0.05) in the IBA, ET, LT and POST squirrels, respectively. CAT level during IBA increased by 22.4% (*p* < 0.05) compared with that during SA. The GPx1 protein remained relatively stable during hibernation compared with the SA group. Interestingly, the four antioxidant proteins in the LT squirrels decreased compared with levels in the IBA and ET squirrels, but there was no significant difference with the SA group. In the POST group, the expressions of SOD1 and SOD2 were at higher levels, whereas the expressions of CAT and GPx1 recovered to SA and PRE levels (figure 3*a*,*b*).

In the liver tissue, compared with the SA group, the expression of SOD1 increased significantly in the IBA and ET groups (83.6%, *p* < 0.05; 76.5%, *p* < 0.05, respectively); SOD2 expression increased by 38.8% (*p* < 0.05) during IBA; CAT expression was at higher levels throughout the entire hibernation period, and increased by 47.9% (*p* < 0.001), 25.1% (*p* < 0.05), 15.9% (*p* > 0.05) and 10.3% (*p* > 0.05) in the IBA, ET, LT and POST groups, respectively; the expression of GPx1 in the IBA group increased significantly by 42.4% (*p* < 0.05) compared with that in the SA group. In addition, except for SOD1, the antioxidant protein expression also increased in the IBA and ET groups compared with the PRE group. However, compared with the IBA and ET groups, expressions in the LT group were downregulated, as observed in the heart tissue. Moreover, the levels of antioxidant proteins recovered to SA and PRE levels after hibernation (figure 3*c*,*d*).

In the brain tissue, SOD1 expression in the IBA and ET groups increased significantly compared with that in the SA group (72.0%, *p* < 0.05; 74.4%, *p* < 0.01, respectively) and PRE group (47.1%, *p* < 0.05; 49.2%, *p* < 0.05, respectively). Compared with the SA group, SOD2 expression increased significantly only in the POST group (65.2%, *p* < 0.05). CAT expression in the brain remained relatively steady during the hibernation period. GPx1 expression in the IBA group increased significantly compared with that in the SA group (37.0%, *p* < 0.01) and increased significantly in the IBA and ET groups compared with that in the PRE group (41.6%, *p* < 0.01; 27.7%, *p* < 0.05, respectively). Overall, the expressions of the four proteins in the LT group showed declining trends compared with the expressions in the IBA and ET groups. Furthermore, with the exception of SOD2, all protein levels were restored to SA and PRE levels after hibernation (figure 3*e*,*f*).

In the kidney tissue, compared with the SA group, SOD1 expression in the IBA group demonstrated a non-significant increase (*p* > 0.05) but showed a significant increase in the ET group (27.5%, *p* < 0.05). SOD1 expression in the PRE group was slightly lower than that in the SA group, but expression in the IBA and ET groups increased significantly (*p* < 0.01) compared with the PRE level. Overall, SOD2 expression was similar to that of SOD1 expression. The expressions of CAT in the IBA and ET groups increased significantly compared with that in the SA group (32.0%, *p* < 0.05; 26.5%, *p* < 0.05, respectively) and PRE group (41.1%, *p* < 0.01; 35.5%, *p* < 0.05, respectively). GPx1 expression in the IBA and ET groups increased significantly compared with that in the SA group (43.7%, *p* < 0.05; 43.5%, *p* < 0.05, respectively) and PRE group (37.0%, *p* < 0.05; 36.8%, *p* < 0.05, respectively). The same as other tissues, the antioxidant proteins in the LT group decreased compared with those in the IBA and ET groups. Moreover, most expressions recovered to SA and PRE levels after hibernation (figure 3*g*,*h*).

### 3.4. Increased antioxidant enzyme activity during interbout arousal and early torpor but decreased activity during late torpor in Daurian ground squirrels

To further explore the effects of antioxidant enzymes on oxidative stress, the activities of SOD1, SOD2, CAT and GPx, as well as TAC, were determined.

**Figure 4.**
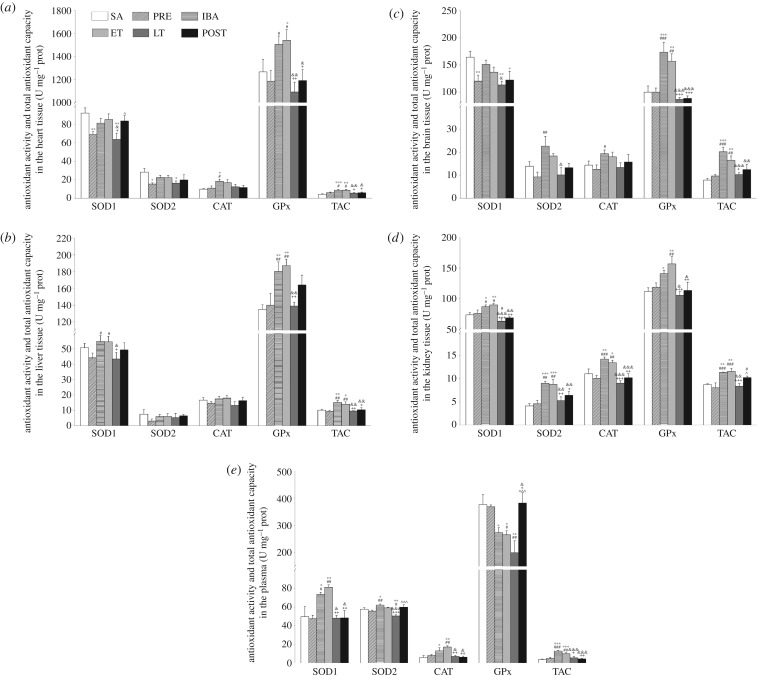
Changes in activities of SOD1, SOD2, CAT, GPx1 and TAC in different tissues of *S. dauricus* over the course of a torpor–arousal cycle. (*a*) Activities of SOD1, SOD2, CAT, GPx and TAC in heart tissue during six hibernation periods. (*b*) Activities of SOD1, SOD2, CAT, GPx and TAC in liver tissue during six hibernation periods. (*c*) Activities of SOD1, SOD2, CAT, GPx and TAC in brain tissue during six hibernation periods. (*d*) Activities of SOD1, SOD2, CAT, GPx and TAC in kidney tissue during six hibernation periods. (*e*) Activities of SOD1, SOD2, CAT, GPx and TAC in plasma during six hibernation periods. SA, summer active; PRE, pre-hibernation; IBA, interbout arousal; ET, early torpor; LT, late torpor; POST, post-hibernation. Values are means ± s.e., *n* = 8; *p < 0.05, compared with SA; **p < 0.01, compared with SA; ***p < 0.001, compared with SA; ^#^*p* < 0.05, compared with PRE; ^##^*p* < 0.01, compared with PRE; ^###^*p* < 0.001, compared with PRE; ^&^*p* < 0.05, compared with IBA; ^&&^*p* < 0.01, compared with IBA; ^&&&^*p* < 0.001, compared with IBA; ^+^*p* < 0.05, compared with ET; ^++^*p* < 0.01, compared with ET; ^+++^*p* < 0.001, compared with ET; ^^^*p* < 0.05, compared with LT.

In the heart tissue, SOD1 and SOD2 activities decreased significantly in the PRE group (25.2%, *p* < 0.01; 45.7%, *p* < 0.05, respectively) and LT group (30.9%, *p* < 0.01; 42.3%, *p* < 0.05, respectively) compared with the SA group. The activities of SOD1 and SOD2 were higher in the IBA and ET groups than in the PRE and LT groups. The activity of CAT in the IBA group increased significantly (87.5%, *p* < 0.05; 68.3%, *p* < 0.05, respectively) compared with that in the SA and PRE groups, with no significant differences found among the other groups. GPx activity in the ET group increased significantly (21.6%, *p* < 0.05) compared with that in the SA group, and increased significantly in the IBA and ET groups (26.9%, *p* < 0.05; 29.9%, *p* < 0.05, respectively) compared with that in the PRE group. Results also showed that TAC in the IBA and ET groups increased significantly compared with that in the SA (*p* < 0.01) and PRE groups (*p* < 0.05) (figure 4*a*).

In the liver tissue, SOD1 activity in the IBA and ET groups increased significantly (24.2%, *p* < 0.05; 23.7% *p* < 0.05, respectively) compared with that in the PRE group. GPx activity increased markedly in the IBA and ET groups compared with that in the SA group (33.6%, *p* < 0.01; 38.7%, *p* < 0.01, respectively) and PRE group (28.7%, *p* < 0.01; 33.7%, *p* < 0.01, respectively). There were no significant differences in SOD2 and CAT activities among the six groups. SOD1 and GPx activities in the LT group were considerably lower (*p* < 0.05 and *p* < 0.01, respectively) than the activities in the IBA and ET groups. Furthermore, TAC showed the same trend for the various antioxidant enzymes, and substantially increased in the IBA and ET groups but decreased in the LT group (figure 4*b*).

In the brain tissue, SOD1 activity in the PRE, LT and POST groups decreased significantly compared with that in the SA group (26.8%, *p* < 0.01; 31.1%, *p* < 0.01; 25.6%, *p* < 0.05, respectively). The activities of SOD2 and CAT in the IBA group were markedly higher than the activities in the PRE group (144.4%, *p* < 0.01; 53.9%, *p* < 0.01, respectively). GPx activity in the IBA and ET groups increased substantially compared with that in the SA group (73.5%, *p* < 0.001; 56.8%, *p* < 0.01, respectively). Compared with the PRE group, GPx activity in the IBA and ET groups increased substantially (73.5%, *p* < 0.001; 56.8%, *p* < 0.01, respectively), whereas GPx activity in the LT and POST groups decreased significantly (*p* < 0.001) compared with that in the IBA and ET groups. In addition, TAC in the IBA and ET groups increased markedly compared with that in the SA group (155.6%, *p* < 0.001; 108.9%, *p* < 0.01, respectively) and was substantially higher than that in the PRE group (figure 4*c*).

In the kidney tissue, SOD1 activity in the IBA and ET groups increased significantly compared with that in the SA group (18.0%, *p* < 0.05; 21.9%, *p* < 0.01, respectively). SOD1 activity in the IBA and ET groups increased significantly compared with activity in the PRE group (14.4%, *p* < 0.05; 18.2%, *p* < 0.05, respectively). SOD1 activity was lowest during LT over the whole hibernation period. SOD2 activity in the IBA and ET groups increased significantly compared with that in the SA group (118.5%, *p* < 0.001; 113.3%, *p* < 0.001, respectively) and PRE group (96.9%, *p* < 0.01; 92.2%, *p* < 0.01, respectively). In addition, SOD2 activity in the LT group decreased significantly (*p* < 0.01) compared with that in the IBA and ET groups. CAT activity in the IBA and ET groups increased significantly compared with activity in the SA group (28.6%, *p* < 0.01; 21.5%, *p* < 0.05, respectively) and PRE group (41.2%, *p* < 0.001; 33.4%, *p* < 0.01, respectively). GPx activity in the IBA and ET groups increased markedly compared with that in the SA group (26.1%, *p* < 0.05; 40.4%, *p* < 0.01, respectively) and increased significantly in the ET group compared with that in the PRE group (32.0%; *p* < 0.01). In the LT group, however, CAT and GPx activities decreased to their lowest levels over the course of the whole torpor cycle. Furthermore, TAC significantly increased in the IBA and ET groups relative to the SA (*p* < 0.01) and PRE (*p* < 0.001) groups and was lowest during LT. In the kidney, all changed indicators recovered to SA or PRE levels after hibernation (figure 4*d*).

In plasma, SOD1 activity in the IBA and ET groups increased significantly compared with that in the SA group (48.1%, *p* < 0.05; 63.5%, *p* < 0.01, respectively) and PRE group (54.8%, *p* < 0.05; 70.9%, *p* < 0.01, respectively). SOD2 activity in the IBA group increased relative to the SA group (7.6%, *p* < 0.05) and PRE group (11.5%, *p* < 0.01). Compared with the SA, PRE, IBA and ET groups, SOD2 activity in the LT group decreased by 12.6% (*p* < 0.01), 9.4% (*p* < 0.05), 18.8% (*p* < 0.001) and 14.7% (*p* < 0.001), respectively. CAT activity in the IBA and ET groups increased significantly compared with activity in the SA group (116.1%, *p* < 0.05; 184.1%, *p* < 0.01, respectively). In addition, CAT activity in the ET group significantly increased by 113.6% (*p* < 0.01) compared with that in the PRE group. GPx activity in the IBA, ET, and LT groups decreased significantly compared with activity in the SA group (27.4%, *p* < 0.05; 29.4%, *p* < 0.05; 46.9%, *p* < 0.01, respectively) and decreased significantly in the ET group (27.8%, *p* < 0.05) and LT group (45.7%, *p* < 0.01) compared with that in the PRE group. Furthermore, TAC in the IBA and ET groups increased markedly (*p* < 0.01) compared with that in the SA and PRE groups (figure 4*e*).

### 3.5. Transcriptional regulation of antioxidant responses in hibernating Daurian ground squirrels

**Figure 5.**
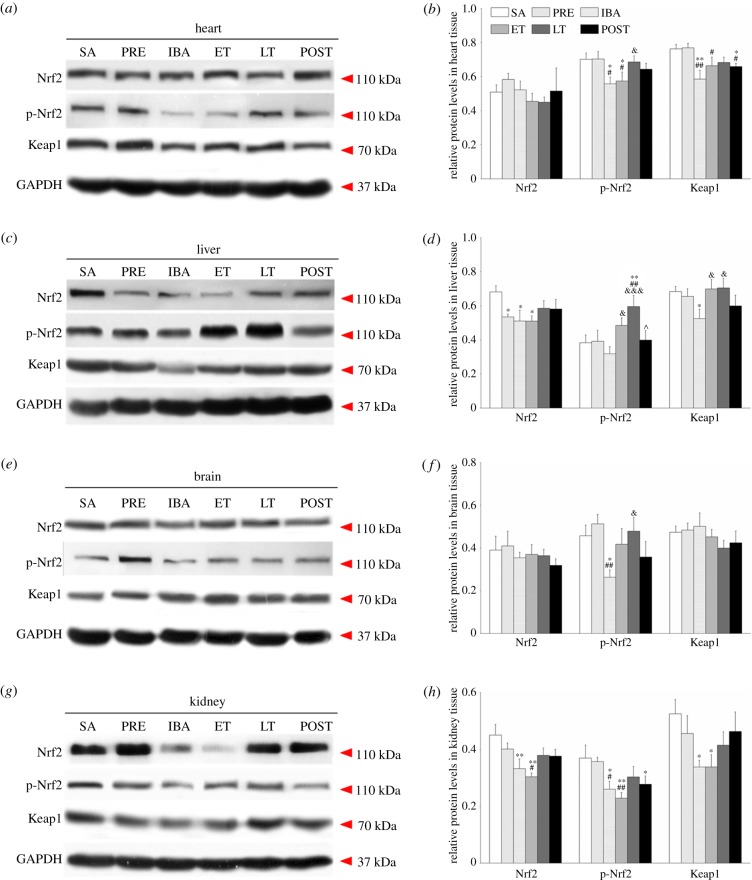
Changes in levels of Nrf2, p-Nrf2 and Keap1 proteins in various tissues during different periods. (*a*) Representative immunoblots of Nrf2, p-Nrf2 and Keap1 in heart tissue during six hibernation periods. (*b*) Relative Nrf2, p-Nrf2 and Keap1 protein expression in heart tissue. (*c*) Representative immunoblots of Nrf2, p-Nrf2 and Keap1 in liver tissue during six hibernation periods. (*d*) Relative Nrf2, p-Nrf2 and Keap1 protein expression in liver tissue. (*e*) Representative immunoblots of Nrf2, p-Nrf2 and Keap1 in brain tissue during six hibernation periods. (*f*) Relative Nrf2, p-Nrf2 and Keap1 protein expression in brain tissue. (*g*) Representative immunoblots of Nrf2, p-Nrf2 and Keap1 in kidney tissue during six hibernation periods. (*h*) Relative Nrf2, p-Nrf2 and Keap1 protein expression in kidney tissue. SA, summer active; PRE, pre-hibernation; IBA, interbout arousal; ET, early torpor; LT, late torpor; POST, post-hibernation. Values are means ± s.e., *n* = 8; *p < 0.05, compared with SA; **p < 0.01, compared with SA; ^#^*p* < 0.05, compared with PRE; ^##^*p* < 0.01, compared with PRE; ^&^*p* < 0.05, compared with IBA; ^&&&^*p* < 0.001, compared with IBA; ^^^*p* < 0.05, compared with LT.

The Nrf2/Keap1 pathway plays a central role in the regulation of antioxidant defence in hibernators [24]. To explore the influence of hibernation-induced oxidative stress on the Nrf2/Keap1 pathway over the course of the torpor–arousal cycle, we determined the expressions of Nrf2, p-Nrf2 (phosphorylated Nrf2) and Keap1 in various tissues by western blotting analysis (figure 5).

In the heart tissue, there were no significant differences in the expression of Nrf2 among all groups. However, the levels of p-Nrf2 decreased significantly in the IBA and ET groups compared with levels in the SA group (20.5%, *p* < 0.05; 18.2%, *p* < 0.05, respectively) and PRE group (20.7%, *p* < 0.05; 18.4%, *p* < 0.05, respectively). Furthermore, Keap1 expression decreased markedly in the IBA and POST groups compared with the SA group (23.3%, *p* < 0.01; 13.7%, *p* < 0.05, respectively) and PRE group (23.9%, *p* < 0.01; 14.3%, *p* < 0.05, respectively). However, the expressions of Nrf2 and p-Nrf2 recovered to SA levels in the POST group (figure 5*a*,*b*).

In the liver tissue, Nrf2 expression decreased significantly in the PRE, IBA and ET groups compared with the SA group (21.6%, *p* < 0.05; 25.1%, *p* < 0.05; 25.2%, *p* < 0.05, respectively). Furthermore, the levels of p-Nrf2 increased significantly in the LT group compared with levels in the SA, PRE and IBA groups (55.4%, *p* < 0.01; 52.1%, *p* < 0.01; 87.1%, *p* < 0.001, respectively). Keap1 expression in the IBA group decreased markedly (23.3%, *p* < 0.05) compared with the SA group. The expressions of the three proteins recovered to SA levels in the POST group (figure 5*c*,*d*).

In the brain tissue, no significant changes were observed in the expressions of Nrf2 or Keap1 among the six periods. The levels of p-Nrf2 in the IBA group decreased significantly compared with that in the SA group (42.4%, *p* < 0.05) and PRE group (48.7%, *p* < 0.01). The expressions of p-Nrf2 recovered to normal levels in the POST group (figure 5*e*,*f*).

In the kidney tissue, Nrf2 expression decreased significantly in the IBA and ET groups compared with the SA group (26.2%, *p* < 0.01; 32.6%, *p* < 0.01, respectively). The levels of p-Nrf2 significantly decreased in the IBA, ET and POST groups compared with the SA group (29.6%, *p* < 0.05; 38.2%, *p* < 0.01; 24.9%, *p* < 0.05, respectively), and in the IBA and ET groups compared with the PRE group (27.2%, *p* < 0.05; 36.1%, *p* < 0.01, respectively). Keap1 expression decreased significantly in the IBA and ET groups compared with the SA group (35.6%, *p* < 0.05; 35.5%, *p* < 0.05, respectively). However, Nrf2 and Keap1 expression recovered to SA levels in the POST group (figure 5*g*,*h*).

## 4. Discussion

This is the first report on the remarkable controllability of oxidative stress and tissue specificity in major oxidative tissues of a hibernating species. Almost all increased or decreased indicators in the tested tissues recovered to SA or PRE levels after hibernation. The Nrf2/Keap1 signalling pathway showed a possible regulating effect on the antioxidant enzyme system in response to potential oxidative stress during the hibernating process; moreover, the changing patterns of the antioxidant enzymes were tissue specific.

There were no significant differences in organ weights among the different periods (figure 1*a*), suggesting that the organs of the adult ground squirrels did not exhibit weight differences with the length of hibernation time. However, compared with the SA group, the organ wet weight/body weight ratio showed significant decreases in the PRE, IBA, ET and LT groups, though not the POST group (figure 1*b*). The main reason for the decrease in this ratio may be that the ground squirrels experienced a period of fattening before hibernation [1,36]. Body weight increased significantly in the PRE group but the weight of the organs did not change, thus the organ-to-body ratio decreased. Body weight decreased significantly until after hibernation, and therefore the organ-to-body ratio recovered to normal levels after hibernation.

H_2_O_2_ is a significant ROS generated due to incomplete reduction of molecular oxygen in the electron transport chain of mitochondria and is a by-product of mitochondrial respiration [8,9,37]. In our study, H_2_O_2_ content significantly increased during LT (when the squirrel entered torpor for at least 5 days and exhibited a stable Tb of 5°C–8°C), especially in the heart and brain (figure 2*a*). Entrance into torpor is characterized by extreme reductions in blood flow, heart rate, oxygen consumption and mitochondrial respiration [2]; for example, oxygen consumption has been shown to decrease by more than 90% during hibernation in Arctic ground squirrels [38]. Long-term hypothermia, hypoxia and ischaemia are considered stressful conditions. Previous studies have shown that ROS production increases during environmental hypoxia [39] and ischaemic anoxia [40], and concentrations vary with stress intensity. In addition, the heart and brain, which contain a great deal of mitochondria and are the main areas of ROS production, appear to be more susceptible to oxidative stress due to hypoxic ischaemia during hibernation [32,41]. Therefore, changes in H_2_O_2_ content in the heart and brain in the LT group were more obvious than changes in the SA or PRE group. However, initiation of arousal from torpor is accompanied by the prompt recovery of physiological functions. Previous research has shown oxygen consumption in squirrels at arousal to be 3-fold higher than that during the active state and 36-fold higher than that during the torpid state [4]; furthermore, liver mitochondrial respiration in ground squirrels has been found to rapidly increase (70%) at arousal relative to that during torpor [42]. The rate of ROS synthesis is positively correlated with mitochondrial respiration and oxygen consumption [43,44]. Theoretically, ROS levels should be higher during IBA than during other states. Nevertheless, in the current study, H_2_O_2_ showed a relatively steady level in the four tissue types during IBA (figure 2*a*), contrary to the above hypothesis. These findings imply that excess ROS may be removed during IBA to protect the organism against oxidative damage. This speculation is supported by studies that show CAT- and SOD-like activities to be noticeably increased in various tissues of Syrian hamsters (*Mesocricetus auratus*) upon arousal [20,21]. As reported previously, hibernators respond to periodic arousal and external stimulation during torpor and arousal cycles [45]. It is possible that excess ROS during LT may prompt squirrel arousal from torpor, and low concentrations of H_2_O_2_ in the IBA state may prevent oxidative damage due to prolonged hibernation. This might be why prolonged hibernation accompanied by low temperature, hypoxia and fasting shows no tissue or cell oxidative damage.

*In vivo*, free radicals undergo lipid peroxidation to form MDA. As the final product of membrane lipid peroxidation and an index of oxidative damage of lipids, MDA can cause protein, nucleic acid and macromolecule cross-linked polymerization, and can exhibit cytotoxicity. In the present study, the level of MDA increased significantly in the LT group compared with that in the IBA group (figure 2*b*). These results are consistent with earlier studies. Chauhan *et al*. [16] showed that MDA content in plasma and erythrocyte membranes increased significantly during hibernation compared to that in non-hibernating active black bears (*Ursus americanus*). An important factor that contributes to the generation of ROS in hibernators is the altered composition of lipid reserves, which is necessary to maintain lipid fluidity at low Tb values [7]. For optimal hibernation, lipid depots must contain elevated levels of polyunsaturated fatty acids (PUFAs), such as linoleic acid [46]; however, PUFAs are very susceptible to free radical attack, leading to autoxidation and the generation of lipid peroxide radicals [47]. Hence, these factors may increase MDA during late hibernation. However, MDA levels during IBA were significantly lower than levels observed during the other states and were consistent with the tendency of H_2_O_2_ in the four tissue types (figure 2*a*). These results, together with the relatively lower H_2_O_2_ levels found during IBA than during torpor, suggest that IBA in hibernators plays a key role in the downregulation of ROS and prevents oxidative damage during torpor.

Oxidative stress is not only associated with increased production of oxidizing species, but also related to significantly decreased antioxidant defence capability. Thus, we further investigated the expression of antioxidant proteins (SOD1, SOD2, CAT and GPx1) in different tissues under different states during hibernation. SODs catalyse the dismutation of 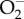
· into H_2_O_2_, with H_2_O_2_ then being decomposed into H_2_O by CAT in the peroxisomes or by GPx in the cytosol [8,37]. In this study, compared with the SA or PRE states, the expression and activity of the four antioxidant enzymes were upregulated in the IBA and ET states in all tissue types tested; however, different antioxidant enzymes also showed differential expression in different tissues throughout the torpor–arousal cycle (figure 3). SOD2 protein levels in the heart increased strongly throughout the entire hibernation cycle, but this change was not very obvious in other tissues; for example, SOD2 expression in the brain only showed a significant increase in the POST group and remained at relatively steady levels in the other stages. Previous study on the skeletal muscle of *S. tridecemlineatus* found that SOD2 protein levels increased strongly in early torpor and remained elevated throughout torpor and arousal [48]. This indicates that SOD2 plays a major role in heart and skeletal muscle with high oxygen consumption protection. In the present study, however, CAT protein expression in the liver significantly increased during the hibernation period, whereas levels in the brain showed no obvious changes. One possible reason is that CAT is found at high concentrations in liver tissue. Furthermore, elevated CAT levels can scavenge H_2_O_2_ induced by hibernation and thus maintain a steady level of H_2_O_2_. This indicates that CAT plays a considerable role in liver protection. In the kidney, the four antioxidant proteins increased significantly during IBA and ET, but we did not find any differences among the various antioxidant proteins. In addition, the activities of the various antioxidant enzymes were higher in the IBA and ET groups than those in the LT group in all tissues. Another important factor that influences enzyme activity is temperature. During IBA, the Tb rapidly recovered to normal (35°C–38°C), which is the optimum reaction temperature for the enzymes. Nevertheless, GPx activity in the plasma decreased significantly during the hibernation season. This decrease may be the result of insufficient transportation of glutathione, a substrate necessary for GPx reactions in the plasma, due to torpor. It is worth noting that most changes in the indicators recovered to SA or PRE levels after hibernation. The torpor–arousal cycle is accompanied by marked reductions in oxygen delivery in torpor and rapid reperfusion of oxygen and blood to all tissues during interbout arousal [2,5]. In response to such physiological and metabolic changes, adequate antioxidant defence is needed to sustain cell viability over weeks of torpor and to defend against potential cellular stress during the transition stages of torpor. Indeed, our results, which showed that the protein expressions of SOD1, SOD2, CAT and GPx1, as well as TAC, increased significantly during both IBA and ET, substantiated this conclusion. Previous studies have also shown that various antioxidant enzymes and low molecular weight antioxidants significantly increase during hibernation [7,19–24]. For example, the expressions of most antioxidant proteins (SOD1, SOD2, GPx1, CAT, NQO1 and Trx2) were upregulated in *Myotis ricketti* bats during arousal [24] and antioxidant proteins and low molecular weight antioxidants (e.g. glutathione, ascorbate) were significantly higher during IBA and ET in *Urocitellus parryii* [38], *Mesocricetus auratus* [49] and *Rhinolophus ferrumequinum* [24]. Upregulation in the expression and activity of antioxidant proteins can catalyse the decomposition of ROS (H_2_O_2_) to H_2_O and O_2_. Consistently, we observed significantly lower levels of H_2_O_2_ and MDA during IBA and ET states. With decreased levels and activities of antioxidant proteins, we found higher levels of H_2_O_2_ accumulation in the LT state (figures 2–4). Therefore, the intracellular antioxidant enzyme system played a key role in fighting oxidative stress during hibernation; moreover, the expression of different antioxidant enzymes displayed tissue specificity. The tissue-specific responses of different antioxidant enzymes may be caused by distinct oxygen consumption and susceptibility to oxidants, and the physiological status of different tissues during hibernation. In the present study, TAC was increased significantly during IBA in all tested tissues. These findings suggest that the upregulation of various antioxidant enzymes during IBA is likely responsible for preventing oxidative damage of tissues.

The transcription factor Nrf2 plays a vital role in maintaining cellular homeostasis, especially upon exposure of cells to oxidative stress, through its ability to regulate the basal and inducible expression of a multitude of antioxidant proteins, detoxification enzymes and xenobiotic transporters [25,26]. Our study showed that the expression of p-Nrf2 decreased by varying degrees in the four tissues during IBA compared with that during SA and PRE but increased significantly during LT in all four tissue types relative to that expressed during IBA and ET, especially in the liver. The Keap1 expression trend was consistent with that of p-Nrf2. Under basal conditions, Nrf2 is sequestered in the cytosol. However, in response to oxidative stress, Nrf2 is released from Keap1, leading to phosphorylation and translocation into the nucleus where phospho-Nrf2 heterodimerizes with small Maf proteins and binds to the ARE, activating the expression of a battery of cytoprotective genes [29,50]. The increase in ROS (H_2_O_2_) during LT may induce excessive pro-oxidant substances, which could activate the Nrf2/Keap1 signalling pathway and increase p-Nrf2 expression. Previous studies on non-hibernators have reported that over-expression of Nrf2 in astrocytes confers protection to neurons from oxidative stress [51], and supplementation with docosahexaenoic acid and extra virgin olive oil may improve liver pathogenic alterations through Nrf2 activation and upregulation [52,53]. In thirteen-lined ground squirrels, the expression of Nrf2 has been shown to increase in heart, liver and brown adipose tissue during hibernation [7,54]. Our finding of decreased p-Nrf2 during IBA and ET in hibernators is contrary to previous research, which reported increased p-Nrf2 expression [7,24]. This may be because in the IBA and ET states, with the increase in antioxidant protein expression and activity in tissues, ROS content was reduced, which, in turn, inhibited the activation and nuclear translocation of Nrf2. The content of ROS during LT increased due to hypoxic ischaemia and reperfusion activated the Nrf2 signalling pathway, which upregulated the expression of downstream antioxidant enzymes during IBA. Therefore, activation of the Nrf2 signalling pathway plays an important role in regulating antioxidant enzymes and removing excess ROS.

In summary, under repeated torpor–arousal cycles, almost all increased and decreased indicators of oxidative stress and antioxidants in the tested tissues recovered to SA or PRE levels after hibernation, suggesting remarkable controllability of oxidative stress in hibernators. In addition, different tissues exhibited different levels of oxidative stress. Elevated H_2_O_2_ content in the heart and brain tissue during LT was observed, implying that these tissues are more susceptible to the effects of oxidative stress. Furthermore, the Nrf2/Keap1 signalling pathway in the Daurian ground squirrel showed a possible regulating effect on the antioxidant enzyme system in response to potential oxidative stress induced during hibernation. The expression of p-Nrf2 decreased significantly in all tissues during IBA but increased significantly during LT in all four tissue types. In addition, the changing patterns of the antioxidant enzymes were tissue specific. Markedly increased SOD1 and GPx1 expressions as well as TAC were observed during IBA and ET in all tissues, suggesting this to be a common mechanism in tissues for protection against oxidative damage over repeated hypoxic ischaemia and reperfusion during torpor-arousal cycles. Elevated SOD2 in the heart and CAT expression in the liver throughout the entire hibernation season are another important mechanism of antioxidant damage. Overall, we showed that increased ROS during LT activated the Nrf2/Keap1 antioxidant pathway, upregulated various antioxidant enzymes during IBA and ET, and likely contributed to the decreased MDA levels during IBA and ET, thereby providing protection from oxidative damage over the torpor–arousal cycle in hibernating Daurian ground squirrels.

## Supplementary Material

A list of antibodies

## Data accessibility

This article has no additional data.
