# Supplementary material for: Controllable oxidative stress and tissue specificity in major tissues during the torpor–arousal cycle in hibernating Daurian ground squirrels
Source: Open Biol. 2018 Oct 10;8(10):180068. doi: 10.1098/rsob.180068 (PMC6223210; doi:10.1098/rsob.180068)
Supplement: A list of antibodies [file rsob180068supp1.docx]

**S1 Table. Antibodies used in this study**

| **Protein Name** | **Product No.** | **Company** | **Polyclonal or Monoclonal** | **Dilution** | **Sample loading/ lane** |
| --- | --- | --- | --- | --- | --- |
| SOD1 | ab13498 | Abcam Corp. | Polyclonal | 1:5000 | 20 μl |
| GPX1 | ab22604 | Abcam Corp. | Polyclonal | 1:1000 | 20 μl |
| Nrf2 | ab137550 | Abcam Corp. | Polyclonal | 1:500 | 20 μl |
| Nrf2 (phospho S40) | ab76026 | Abcam Corp. | Monoclonal | 1:5000 | 20 μl |
| Keap1 | ab139729 | Abcam Corp. | Polyclonal | 1:1000 | 20 μl |
| SOD2 | 13141S | Cell Signaling Technology | Polyclonal | 1:1000 | 20 μl |
| CAT | 14097S | Cell Signaling Technology | Polyclonal | 1:1000 | 20 μl |
